# Holidays Abroad and the Eating Behavior of the Inhabitants of Poland

**DOI:** 10.3390/ijerph192315439

**Published:** 2022-11-22

**Authors:** Anna Katarzyna Mazurek-Kusiak, Agata Kobyłka, Natalia Korcz, Andrzej Soroka

**Affiliations:** 1 Department of Tourism and Recreation, University of Life Sciences in Lublin, Akademicka 13, 20-950 Lublin, Poland; 2 Department of Natural Foundations of Forestry, Institute of Soil Science and Environment Management, University of Life Sciences in Lublin, Akademicka 13, 20-950 Lublin, Poland; 3 Institute of Health Sciences, Siedlce University of Natural Sciences and Humanities, 2 Konarskiego Street, 08-110 Siedlce, Poland

**Keywords:** all inclusive, meals, restaurant, diet, hotel, breakfast, dinner, choice, factors

## Abstract

A hotel is interested that the guest buys from it not only accommodation, but also catering services, preferably an all-inclusive option. However, many tourists choose only accommodation or accommodation with breakfast, and dinners and other things are purchased outside the place of accommodation. Therefore, it is important to know the eating behavior of tourists, and what hotels must do to make guests want to use food services at the place of accommodation. The purpose of this article is to show the reasons for not buying full meals at hotels during vacations by the inhabitants of Poland. The study used the diagnostic survey method with the help of the direct survey technique. A proprietary survey questionnaire was developed. The direct survey was conducted among 3071 tourists across the country. The study was conducted in 2019–2020. For data analysis, a discriminant function was chosen to examine the differences between groups based on a set of selected independent variables. When buying tourist holidays in travel agencies, 32.40% of Poles bought the all-inclusive option, 33.15% bought breakfast and dinner, 12.47% bought breakfast only, while 21.98%, bought accommodation without any food. For tourists who did not buy any meals at the hotel, the most important factors for eating out were mainly unwillingness to adapt to the hours of serving meals at the place of accommodation, and the desire to control the quality of raw materials needed for preparation of individual dishes. Among hotel guests who only had breakfast at the hotel, the main reasons for eating lunch and dinner outside of the hotel were the desire to try local dishes in regional restaurants, to get to know different restaurants, and to eat meals made entirely of ecological materials. A big barrier to buying meals in a hotel was the lack of offering dietetic dishes or their too high price. Older people dined out because of the lack of dietary dishes or their too high price and because they look for restaurants that serve meals prepared from ecological ingredients. Younger people, on the other hand, did not dine at the hotel because they did not want the hours of serving meals at the hotel to limit their sightseeing in the city and surroundings.

## 1. Introduction

Processes taking place in the environment of the contemporary consumer such as globalization, economic and demographic changes, changing lifestyles, and a more comprehensive range of products and services, cause changes in consumption models and the emergence of new consumer behaviors.

Consumers’ behavior in the hotel and catering services market is also changing. In the era of intense market competition, satisfying basic nutritional and accommodation needs are not an acceptable way to attract hotel customers. The nutritional behavior of tourists mainly concerns places of consumption and frequency of consumption [1]. Modern tourists have growing requirements and expect better and better services in terms of nutrition. While consuming food products, they expect to satisfy hunger and thirst and pay attention to such factors as safety, natural ingredients, comfort, high nutritional values, and care for the environment [2,3]. Moreover, among the trends in the behavior of modern consumers in the hotel and catering services market, it is worth particularly emphasizing the increased awareness of the impact of selected food on health, searching for offers that meet individual needs, appreciating new sensations and experiences, virtualization and computerization of consumption [4,5]. An increasing group of hotel guests is also interested in environmental impacts, local products and products dedicated to people with special diets [6,7,8].

It should be remembered that the menu is a kind of representation of the chef’s vision and philosophy. The card should be composed in a way that, despite the limited choice, everyone can find something for themselves. An ideal menu card must be consistent and legible, and correspond to a given place. Dishes must be varied in terms of calorific value, price, and guests’ preferences. Children, vegetarians, and people with food allergies should be considered. The most critical challenge in creating a hotel menu is matching guests’ culinary tastes worldwide [3,9]. The price of the offered dishes is also significant. Therefore, the menu card, in addition to traditional dishes, should include dishes with reduced energy values and reduced content of fat and cholesterol, sodium, sucrose, and simple sugars. More dishes should be enriched with vitamins, minerals, and dietary fibre. The hotel should serve special dishes for people who are on a low-calorie diet, as in [2].

The hotel would like the guest to buy accommodation and catering services, preferably an all-inclusive option [10,11]. However, many tourists choose the accommodation or bed and breakfast themselves and buy lunches and services outside the accommodation. The research by Goswami et al. [12]. showed that in India only 39% of hotel guests eat in hotel restaurants, 43% of visitors do so in Turkey according to Zorlu et al. [13], while in Croatia the percentage is 45% [14].Therefore, it is essential to know the eating behavior of tourists so that hotels know what to do to attract guest to their catering services at the hotel. This article aims to show why inhabitants of Poland do not buy full meals at the hotel during holidays. The purpose of the article was to determine the eating behavior of tourists during trips organized by travel agencies. Therefore, the following research questions were posed in this study:

RQ1: What types of food do tourists eat during organized travel trips?

RQ2: What are the reasons that tourists with different demographic characteristics and eating habits do not purchase a full meal package?

In addition, the research hypothesis was that the reasons for not buying full meals at the hotel differ by age, gender, type of residence and diet of the respondents.

## 2. Literature Review

Food consumption constitutes an integral element of a holiday experience [15]. The eating behavior of hotel guests is an essential element of tourism from the point of view of creating demand and expenses for catering services. The development of gastronomy and tourism is geared toward two audiences: the tourist who buys ordinary products and those that buy luxury products. Mass tourism encourages the development of gastronomic facilities offering so-called fast food. But alongside this there is a market of gourmets traveling in the spirit of slow tourism, who are not in a hurry and want to see and eat something special and unusual [16,17]. According to Calveras [10], tourist holidays with the all-inclusive option do not always appeal to tourists. Tourists expect new experiences in the field of food. This manifests itself, among other things, in the choice of food (preferences as to local dishes), changes in cultural practices (participation in local rituals), and the emergence of new beliefs (acceptance of local symbolism) [18,19,20].

An important element is the high quality of meals, the way the dishes are served, including taste experience, the composition of the menu, the design of the hotel restaurant, the interaction between staff and customers, the atmosphere of the hotel restaurant, the music, and the developed brand [21]. Another important factor is whether the restaurant prepares and serves its meals according to sustainable principles. According to studies by Sarmiento and Hanandeh [22] and Karagiannis and Andrinos [23], hotel vacationers using restaurants are willing to pay more if the restaurant follows sustainable practices. Another important factor is brand. A brand consists of two elements: service credibility and expertise [24]. Service credibility means that the advertised service is the same as the service served and meets the expected standards. As a result, consumers do not look for a restaurant outside the hotel, but choose to stay at a particular hotel because of the “good” restaurants [25]. Expertise means meeting all sanitary and food preparation requirements [26].

Customer satisfaction management is the only strategy that can respond to market changes. Corporate marketing has set the primary goal of focusing on customer satisfaction, which, through the customer experience, can increase customer loyalty, and thus contribute to higher demand for hotel restaurant services and higher profitability [27].

Orlowski [16] wrote that meals on vacation are becoming feasts accompanied by music and entertainment, and where one can learn about local folklore. Hotels, therefore, should organize evenings of folklore, local cuisine and the feasting customs of the country.

Basic forms of tourist service, whose only function until recently was to provide tourists with accommodation, food or transportation, are increasingly becoming tourist attractions or even a key element for creating independent tourism products [28,29]. According to Güneş [30], restaurants that offer local and organic food to their customers have greater efficiency and effectiveness in terms of saving water, energy, and cooking with local products, but also attract many more consumers through these measures. Aslimoski and Gerasimoski [31] point out that the role of food as a cultural attraction and the motivation of tourists to visit a tourist destination is more and more often emphasized, not just as a segment of the accommodation area. Contemporary tourists begin the process of voluntary and conscious education necessary to create a group of gourmets for whom the motive of visiting is the gastronomic identity of the location [32]. According to Makałka [33], it is easier for consumers to give up the daily, fast pace of life during holidays and rest, calm down and enjoy the charms of nature. This is also confirmed by a study by Karagiannis and Andrinos [23]. Therefore, according to Brun [34], tourists most often choose meals in places that offer dishes that have good quality in terms of nutritional value and food security, prepared from local raw materials, prepared from ecological products, that are pro-health and dietary.

Therefore, hotels that want to have loyal customers who use the hotel’s restaurant should have an appropriate menu and high-quality meals tailored to the requirements of individual customers, and a distinctive way of serving customers compared to the competition, having a unique decor and atmosphere in the restaurant [35]. This is very important, as the intention to visit again and the willingness to pay a higher price have long been considered key indicators of loyal customer behavior, as well as recommending the hotel to their friends and giving positive reviews on social media and booking portals [36].

## 3. Materials and Methods

The research used the method of a diagnostic survey with the direct questionnaire technique. An original questionnaire was developed. The survey questionnaire consisted of two parts, the first of which was questions about the nutritional behavior of tourists during their holidays. The questions concerned, among others, the type of diet consumed, the type of meals purchased in 3-Star hotels during tourist holidays, and the factors causing failure to purchase entire board at the hotel. The second part contained questions defining the characteristics of the respondents (professional status, sex, age, place of residence, province).

The research was conducted from 2019 to 2020 using the traditional method in the form of direct research. The survey, coding and entering data from survey forms into the EXCEL 2016 spreadsheet was carried out by a company selected by public pricing. The research was financed by the National Science Center as part of the Miniatura 2 competition for scientific activities No. 2018/02/X/HS4/02427.

The direct survey was conducted among 3071 tourists who bought foreign holidays in a travel agency and chose accommodation in 3-Star hotels. The research was conducted throughout Poland. While making efforts to ensure that the research on consumer behavior in the hotel and catering services market was reliable, the selection of the sample was purposeful and quota-based, with the following factors being the control variables: province, gender, type of place of residence (city, village) and age. The structure of the community was defined as 0.01% of the total population of Poland. The study included 47.83% adult men and 52.17% women. Regarding place of residence, the respondents included 61.12% urban residents and 38.88% rural residents. Due to the age of the respondents, 26.08% of the surveyed people were 18–25 years old, 19.70% were 26–35 years old, 15.40% were 36–45 years old 17.56% were 46–55 years old and 21.26% were 55 and more years old (Table 1).

The Statistica 13 PL program was used for statistical calculations. A discriminant function was selected for data analysis, which examined the differences between groups based on a set of selected independent variables, using the formula [37].

(1) $$D_{kj}=\beta_{0}+\beta_{1}x_{1kj}+\cdots +\beta_{p}x_{pkj}$$

where *p* is the number of discriminant variables, *n* is the sample size, *g* is the number of groups, *D_kj_* is the canonical value of the discriminant function for the *k*-th case in the *j*-th group, *k* = 1, …, *n*, *j* = 1, …, *g*, and *x_kj_* is the value of the *i*-th discriminant variable for the *k*-th case in *j*-th group.

Using discriminant analysis, the reasons for not buying a complete meal in a hotel were examined, considering the sex, the type of place of residence, age and diet of the respondents. The primary purpose of the discriminant analysis was to predict the classification of cases. The research used classification functions to calculate their coefficients, which were determined for each segment of tourists. The given case was classified into the group with the highest classification value. Before starting the analyses, the multivariate normality was examined by checking each variable for the normality of the distribution using Kolmogorov-Smirnov, W Shapiro-Wilk, and Lilliefors tests. It was assumed that the variance matrices of variables were homogeneous in groups. Slight variations were not that important due to the large size of the groups. The differences and dependencies were determined statistically significant at *p* <0.05.

## 4. Results

In the first stage of the research, tourists were asked what option of dining in a hotel they chose when buying tourist holidays in a travel agency.

Most of the respondents, as much as 33.15%, purchased breakfast and dinner, and slightly fewer picked the all-inclusive option (32.40%). The next group of respondents purchased holidays without meals in a hotel (21.98%), and breakfast was bought only by 12.47% of the surveyed tourists.

**Figure 1:**
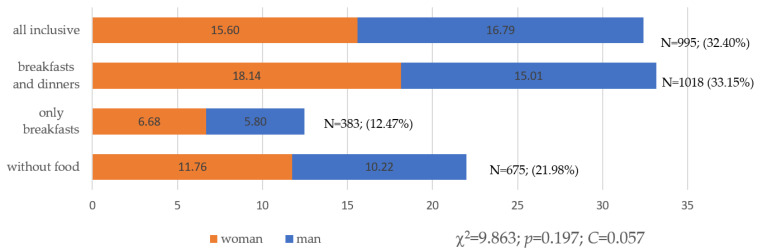
Choice of food and beverage options when purchasing travel vacations from a travel agency by sex (N = 3071). Source: own study based on the research.

**Figure 2:**
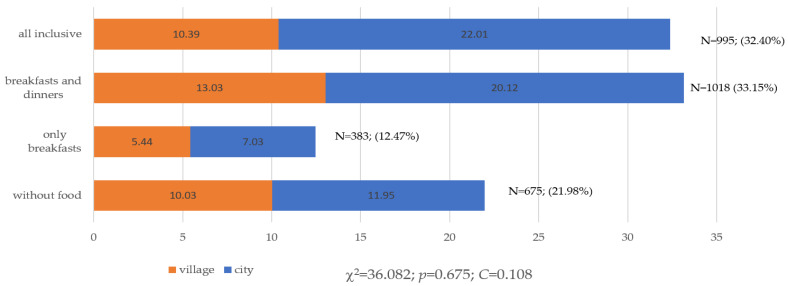
Choice of food and beverage options when purchasing travel vacations from a travel agency by the type of place of residence (N = 3071). Source: own study based on the research.

There were no significant differences in nutritional behaviors regarding the sex of the surveyed tourists (*p* = 0.197; Figure 1) or the type of place of residence (*p* = 0.675; Figure 2).

**Figure 3:**
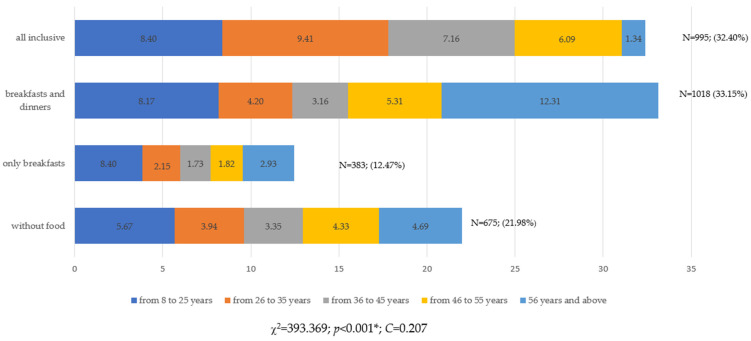
Choice of food and beverage options when purchasing travel vacations from a travel agency by age of respondents (N = 3071). * Statistically significant differences (*p* < 0.05). Source: own study based on the research.

Nutritional behaviors differed significantly due to the age of Poles (*p* < 0.001, χ^2^ = 393.369). The C-Pearson contingency coefficient was 0.204, proving the average degree of correlation (Figure 3).

**Figure 4:**
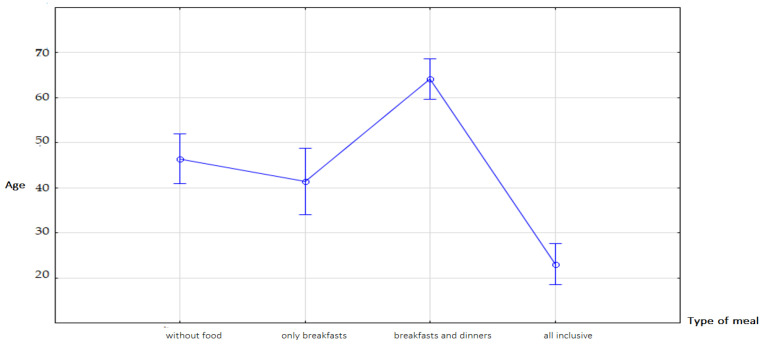
Choosing food options when buying travel vacations from a travel agency F(3.307) = 53.229; *p* < 0.001. Source: own study based on the research.

Most of the youngest people used the all-inclusive option (8.40%), and the oldest people used the breakfast and dinner option (13.31%) (Figure 3). This was confirmed by the Fisher NIR test (Figure 4).

**Figure 5:**
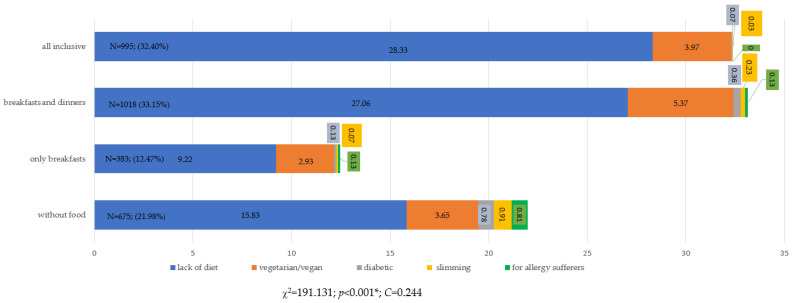
Choice of food and beverage options when purchasing travel vacations from a travel agency by the respondents’ diets (N = 3071). * Statistically significant differences (*p* < 0.05). Source: own study based on the research.

Nutritional behaviors differed significantly due to respondents’ diets (*p* < 0.001, χ^2^ = 191.131). The C-Pearson contingency coefficient was 0.244, showing an average degree of correlation (Figure 5). Those without any diet were most likely to choose the all-inclusive option, vegetarians/vegans were most likely to buy breakfast and lunch at the hotel restaurant, and those with diabetic, slimming and allergic diets were most likely not to use the food at the hotels. It should be noted that none of the respondents with allergies chose the all-inclusive option.

In the second stage of the study, two groups of respondents (without food and only breakfast) were asked about the reasons for not buying a complete meal in a hotel (Table 2, Table 3 and Table 4).

The model of the discriminant function included all the reasons that were subject to assessment. For tourists who did not purchase any meal at the hotel, the essential factors of eating outside the hotel were reluctance to adjust to the hours of serving meals in the hotel (1.558) and the quality of the raw materials used to prepare individual dishes, which was possible thanks to self-preparation of food during tourist trips (0.469). Among hotel guests who only ate breakfast in the hotel, the main reasons for eating lunch and dinner outside the hotel were the desire to try local dishes (1.141), the desire to learn about different restaurants (0.948), the desire to eat meals made of organic ingredients (0.647) and too high a price of dietary meals (1.183) (Table 2). The correlation coefficient between the difference function and the first variable was 0.35 (*p* < 0.001), so the index of the importance of discriminant analysis was average.

All the assessed factors entered the discriminant function model. For personal reasons, the highest discriminatory power, F = 306.776, was achieved by “I like to prepare meals on my own because I know what I eat.” With *p* < 0.001, such a declaration was presented by the highest degree by people aged 26–35 (3.788). These people often go on vacation with young children and want to ensure that they feed their children with healthy meals. The discriminant function F = 107.617 had almost three times lower values because “there are too expensive dietary meals in the hotel” This reason was most important for the oldest people (2016). The high value of the discriminant function F = 80.756 was obtained for the reason “I want to eat meals prepared from organic ingredients.” In this case, most declarations were made by consumers aged 46–55 (1.249). A significant reason, especially for consumers aged 36–45 (1.382), was the desire to taste local food (F = 52.022). Another reason that qualified for the model was “I want to avoid limiting sightseeing with meals in the hotel” (F = 33.524). This reason was critical for the youngest respondents (1.558). The lowest classification power was obtained by the factor “I want to eat in different restaurants.” This factor was also the most significant for respondents aged 18–25 (0.635) (Table 3). The correlation coefficient between the difference function and the first variable was 0.77 (*p* < 0.001), so the validity index of the discriminant analysis indicated a strong relationship between the groups and the discriminant functions. The other correlation coefficients were lower, and were, respectively, 0.58; 0.44; 0.32 (*p* < 0.001). All discriminant functions were significant. As 64% of all discriminatory power was explained by the first function, the second function explained only 22% of all discriminatory power, the third function explained 9%, and the fourth function explained only 5%.

In the case of the classification of respondents according to their diets, there was a weak general discriminatory power in the created model. Only two out of six reasons entered the model. The reason “too expensive dietary meal” was the most significant for people dieting due to allergies (1.296) and people with diabetes (1.221) and “I want to avoid limiting sightseeing with meals at the hotel.” In this case, the cause was most critical for those following the vegetarian/vegan diet (1.048) and least important for those following the diabetic diet (0.500) (Table 4). The relations coefficient between the difference function and the first variable was 0.18, so the discriminant analysis validity index indicated a weak association between groups and discriminant function. Seventy-three percent of all discriminatory power was explained by the first function, the second function explained only 17% of all discriminatory power, the third function explained 7%, and the fourth function explained only 3%.

## 5. Discussion

Few researchers have attempted to analyze this topic. In the articles published so far, there is a lack of work illustrating why tourists do not choose a hotel restaurant at their place of accommodation.

Research by Kowalczuk [38] on the relationship between the way meals are organized during a tourist trip and the demographic, social, and economic characteristics of the respondents showed that gender slightly differentiated the behavior of the respondents in this regard. Only in the case of dinner, he stated that statistically significantly more women do not eat this meal in hotels. We did not show any significant differences in the gender of the respondents in terms of the type of meals purchased in hotels. The research by Kowalczuk [38] has additionally shown that people from villages and smaller towns are more interested in preparing meals on their own. With an increase in the size of the place of residence, the interest in eating meals in the place of accommodation increased. A similar relationship occurred in the case of income growth [26]. Similar results were obtained by Jeżewska-Zycowicz [39], Wolny [40] and Levtska [41]. In our research, the type of place of residence did not have a significant influence on the choice of the place where meals were purchased. According to Kowalczuk [38], older people were less interested in using catering services in hotels than younger consumers, which was confirmed by our research. A greater interest in using catering services in the place of accommodation by young tourists was also found in other studies [41,42].

According to Zimna [43], the barriers to eating meals in hotels are the price and higher energy value with lower nutritional value. This problem was also highlighted by Abramski et al. [1], Kowalczuk [38], and Edwards [44]. Our research confirmed that the barrier to eating a meal in hotels is the price of dietary dishes or the lack of them on offer, the willingness to eat in various restaurants, but also the desire to try local dishes, control of the raw materials from which meals are prepared, and reluctance to limit the visiting time to the hours of serving meals in the hotel. Studies by Chebli & Said [45] and Adamski et al. [1] also showed that hotel guests eat outside the hotel due to the desire to search for new flavors, learn about new culinary novelties, innovative ways of serving dishes, and interesting interior design, which was also confirmed by Nilashia [9] and Kozłowska [46]. From the tourism industry’s point of view, the opportunity to sample high-quality locally produced food products can enhance the tourist’s experience, raise awareness of the region or destination country and encourage first and repeat visits [47].

According to Kowalczuk [38], constant eating times are an incentive to eat meals in a hotel. However, Abramski et al. and Netemeyer et al. [1,35] did not agree with this, showing that hotel guests are not interested in eating meals in the place of accommodation due to the fixed hours of serving meals. Our research results confirm the results Abramski et al. obtained.

In turn, studies by Bondzi-Simpson and Ayeh [48], Kim et al. [8], and Zhang [20] showed that regional, traditional cuisines in middle-developed countries play a crucial role in the organization of festivals, and cultural workshops, but their presence in the menu of hotel gastronomy is insignificant. The same is true with organic products [7]. For this reason, guests dine outside the hotel. We also point out that consumers aged 35–46 dine outside the hotel restaurant to try local dishes. Guests do this mainly during lunch and dinner.

An important aspect is adjusting the menu to the dietary needs of guests. According to the HRS 2020 report [49], on in ten of surveyed hotel guests was currently on a specific diet. Of the respondents, 3% gave up meat, 3% avoided gluten, and 3% ate a vegan diet. Although these are relatively small percentages, the hotel should be prepared for the nutritional limitations of customers. In turn, studies by Cichoń and Bajda [50] show that the presence of vegan equivalents in the breakfast menu was confirmed by 71% of the surveyed hoteliers. As many as 70% of hoteliers declared that their breakfast menu included products for people intolerant to gluten. In comparison, 65% of respondents said lactose-free products could be consumed in their facility. However, these options were lacking in the case of cheese lunches and dinners. Hoteliers said that, on request, the hotel could serve any diet, such as kosher, Mediterranean, Paleo, or a diet individually selected. Still, it was necessary to inform the service in advance and pay an additional fee. Cichoń and Bajda [50] also showed that one in ten hotel guests followed a specific diet. Hotels serve an average of 20 to 80 types of breakfast dishes, but there are also places where you can choose from as many as 100 products. White bread has been replaced by a wide selection of bread, rolls made of various flours, and buns, as well as all kinds of sweets, from croissants to puff pastries. More often, hoteliers also offer gluten-free and lactose-free products for breakfast. Unfortunately, other meals are less likely to include diet options. The offer of dietary dishes is available mainly in hotels in cities, but not in tourist places. In turn, Eren et al., 2021 [51] showed that hotel bosses did not know about allergies. Only 2% of hotels employ dieticians to compose a diet for the sick with allergies, or people dieting. Therefore, the lack of access to dietary meals during lunches and dinners, or their too high price, is a reason for dining outside the hotel restaurant. This was also confirmed by the authors of the article. Beerli-Palacioet et al. [52] recommended that hotels give up the service of a candy tray or a welcome fruit basket in a hotel room in favor of introducing a 24-h diet menu in the hotel restaurant.

## 6. Strengths and Limitations

The results of a very large survey group incliuding representative of the Polish population, were analyzed. The surveys were conducted by a public opinion research company.

The research was conducted only on Poles, whose average monthly income is lower than that of citizens of most European Union countries. The research cannot be applied to all European tourists. Future research should include residents of other European countries with higher monthly incomes. An additional limitation is subjectivity when respondents gave their answers. Respondents in surveys often do not give honest answers.

## 7. Conclusions

The variables differentiating the reasons for not eating or using half-board at a hotel restaurant during vacation travel were age and diet. For tourists who did not purchase any meal at the hotel, the most important reasons for eating outside the hotel were, primarily, reluctance to adjust to the hours of serving meals in the place of accommodation and the desire to control the quality of materials needed to prepare individual dishes.

Among hotel guests who only ate breakfast in the hotel, the main reasons for eating lunch and dinner outside the hotel were to try local dishes in regional restaurants, learn about different restaurants, and eat meals prepared entirely from environmentally friendly ingredients. A significant barrier to buying meals at the hotel was the lack of dietary dishes or their high price.

Older people ate meals outside the hotel due to the lack of dietary dishes or their too high price, and the search for restaurants serving dishes prepared from environmentally friendly ingredients. Younger people, on the other hand, did not dine in the hotel mainly because they did not want the hours of serving meals to limit them while visiting the city and the surrounding area.

Allergy sufferers did not use the hotel restaurant due to the excessive price of dietary dishes, and Vegetarian/Vegetarians did not like the hotel’s serving hours.

Breakfast should attract guests to the hotel restaurant for lunch and dinner, so breakfasts should reflect the local atmosphere and offer dishes based on local delicacies and organic products. In addition, other meals should be advertised during breakfast. Lunch and dinner, on the other hand, should be as varied as breakfast to encourage people with different diets to enjoy them.

## 8. Implications for Practitioners

Hotels should organize folklore evenings, showcase local cuisine and feasting customs of the country, serve organic and dietary meals, while not inflating prices for additional services. At the same time, they should take care to make the decor and atmosphere of the hotel restaurant stand out from the competition. Travel agencies should inform their customers about the types of meals sold in hotel restaurants. Hotels to be competitive in the lodging market can introduce the services of a doctor-dietitian into their offerings.

## Figures and Tables

**Table 1:** General characteristics of the study population.

Specification		Proportion in %
Sex	M	47.83
	F	52.17
Place of residence	villages	38.88
	city	61.12
Age	18–25 years old	26.08
	26–35 years old	19.70
	36–45 years old	15.40
	46–55 years old	17.56
	55 and more years old	21.26
Education	basic	3.29
	vocational	10.68
	secondary	47.83
	higher	38.20
Professional status	working mentally	25.92
	physically working	22.11
	own business	13.55
	freelance	7.91
	manager	5.54
	pupil/student	13.06
	pensioner	9.57
	unemployed	2.34

Source: own study based on the research.

**Table 2:** Factors for not buying full board at the hotel (N = 2013).

Factors	Model of Discriminant Analysis: Wilks’s λ: 0.190; F(24.366) = 92.810; *p* < 0.001;				Classification Functions: A Hotel Guest Who:	
	Wilks’s λ	F	*p*	Tolerance	Did Not Purchase Any food	Purchased Only Breakfast at the Hotel
I don’t want to limit my sightseeing with meals at the hotel	0.889	17.552	<0.001 *	0.875	1.558	1.108
I want to try local dishes	0.889	18.295	<0.001 *	0.877	0.926	1.141
I want to eat in a variety of restaurants	0.905	37.214	<0.001 *	0.860	0.732	0.948
I want to eat meals prepared with organic ingredients	0.878	4.803	0.029 *	0.915	0.294	0.647
I like to prepare meals myself because I know what I’m eating	0.883	11.148	0.001 *	0.912	0.469	0.357
Too expensive dietary meals (vegetarian/vegan diet; diabetic diet; slimming diet, allergic diet)	0.882	9.338	0.003 *	0.910	1.024	1.183
Constant					−5.685	−7.933

* Level of significant difference at *p* < 0.05. Source: Own study based on the research.

**Table 3:** Factors for not buying full board at hotel by age of respondents (N = 2013).

Factors	Model of Discriminant Analysis: Wilks’s λ: 0.190; F(24.366) = 92.810; *p* < 0.001				Classification Functions: Age				
	Wilks’s λ	F	*p*	Tolerance	From 18 to 25 Years	From 26 to 35 Years	From 36 to 45 Years	From 46 to 55 Years	56 Years and Above
I don’t want to limit my sightseeing with meals at the hotel	0.215	33.524	<0.001 *	0.846	1.558	1.108	0.665	0.994	0.988
I want to try local dishes	0.228	52.022	<0.001 *	0.795	0.369	0.077	1.382	0.348	0.520
I want to eat in a variety of restaurants	0.196	8.007	<0.001 *	0.832	0.635	0.245	0.114	0.404	0.532
I want to eat meals prepared with organic ingredients	0.249	80.756	<0.001 *	0.823	0.022	0.356	0.375	1.249	0.071
I like to prepare meals myself because I know what I’m eating	0.413	306.776	<0.001 *	0.854	0.859	3.788	0.649	0.460	0.831
Too expensive dietary meals (vegetarian/vegan diet; diabetic diet; slimming diet, allergic diet)	0.268	107.617	<0.001 *	0.963	1.549	0.632	0.708	1.039	2.016
Constant					−8.573	−11.836	−6.626	−7.576	−8.923

* Level of significant difference at *p* < 0.050. Source: Own study based on the research.

**Table 4:** Factors for not buying full board at the hotel due to respondents’ diets (N = 2013).

Factors	Model of Discriminant Analysis: Wilks’s λ: 0.190; F(24.366) = 92.810; *p* < 0.001				Classification Functions: Residents’ Diet				
	Wilks’s λ	F	*p*	Tolerance	Lack of Diet	Vegetarian/ Vegan	Diabetic	Slimming	For Allergy Sufferers
I don’t want to limit my sightseeing with meals at the hotel	0.968	3.390	0.009 *	0.857	0.944	1.048	0.500	0.947	0.864
I want to try local dishes	0.957	0.368	0.831	0.849	0.736	0.762	0.807	0.609	0.781
I want to eat in a variety of restaurants	0.960	1.140	0.336	0.836	0.328	0.358	0.034	0.176	0.263
I want to eat meals prepared with organic ingredients	0.959	0.872	0.480	0.920	0.486	0.426	0.364	0.334	0.602
I like to prepare meals myself because I know what I’m eating	0.959	0.700	0.592	0.909	1.016	1.103	1.029	0.970	0.988
Too expensive dietary meals (vegetarian/vegan diet; diabetic diet; slimming diet, allergic diet)	0.969	3.661	0.006 *	0.911	1.005	0.923	1.221	1.143	1.296
constant					−5.847	−7.455	−8.181	−8.569	−10.025

* Level of significant difference at *p* < 0.050. Source: Own study based on the research.

## Data Availability

Not applicable.
